# Inhibition of WEE1 Suppresses the Tumor Growth in Laryngeal Squamous Cell Carcinoma

**DOI:** 10.3389/fphar.2018.01041

**Published:** 2018-09-28

**Authors:** Meng-Ling Yuan, Pei Li, Zi-Hao Xing, Jin-Ming Di, Hui Liu, An-Kui Yang, Xi-Jun Lin, Qi-Wei Jiang, Yang Yang, Jia-Rong Huang, Kun Wang, Meng-Ning Wei, Yao Li, Jin Ye, Zhi Shi

**Affiliations:** ^1^Department of Cell Biology – Institute of Biomedicine, National Engineering Research Center of Genetic Medicine, Provincial Key Laboratory of Bioengineering Medicine, College of Life Science and Technology, Jinan University, Guangzhou, China; ^2^Department of Otolaryngology-Head and Neck Surgery, The Third Affiliated Hospital, Sun Yat-sen University, Guangzhou, China; ^3^Department of Urology, The Third Affiliated Hospital, Sun Yat-sen University, Guangzhou, China; ^4^Division of Pulmonary and Critical Care, Department of Internal Medicine, The Third Affiliated Hospital, Sun Yat-sen University, Guangzhou, China; ^5^Department of Head and Neck, Sun Yat-sen University Cancer Center, State Key Laboratory of Oncology in South China, Collaborative Innovation Center for Cancer Medicine, Guangzhou, China

**Keywords:** laryngeal squamous cell carcinoma, WEE1, MK-1775, cell cycle, apoptosis, reactive oxygen species

## Abstract

WEE1 is a tyrosine kinase that regulates G2/M cell cycle checkpoint and frequently overexpressed in various tumors. However, the expression and clinical significance of WEE1 in human laryngeal squamous cell carcinoma (LSCC) are still unknown. In this study, we found that WEE1 was highly expressed in LSCC tissues compared with adjacent normal tissues. Importantly, overexpression of WEE1 was correlated with T stages, lymph node metastasis, clinical stages and poor prognosis of LSCC patients. Furthermore, inhibition of WEE1 by MK-1775 induced cell growth inhibition, cell cycle arrest and apoptosis with the increased intracellular reactive oxygen species (ROS) levels in LSCC cells. Pretreatment with ROS scavenger *N*-acetyl-L-cysteine could reverse MK-1775-induced ROS accumulation and cell apoptosis in LSCC cells. MK-1775 also inhibited the growth of LSCC xenografts in nude mice. Altogether, these findings suggest that WEE1 is a potential therapeutic target in LSCC, and inhibition of WEE1 is the prospective strategy for LSCC therapy.

## Introduction

Laryngeal carcinoma is the second most common malignant tumor of the respiratory system in the male compared to its relative rare in the female (Siegel et al., 2018). The aggressive type of squamous cell carcinoma (LSCC), originating from the laryngeal epithelium, accounts for approximately 90% of laryngeal carcinoma cases (Li et al., 2017). In spite of therapeutic advances, the 5-year survival rate for LSCC patients remains generally unsatisfactory (∼59.6–66.8%) (Hsueh et al., 2017). Thus, it is of great significance to comprehensively understand intrinsic molecular mechanisms underlying LSCC tumorigenesis and identify novel therapeutic targets for the diagnosis and prognostic assessment of LSCC.

WEE1 is a tyrosine kinase that phosphorylates CDC2 at Tyr15 residue and prevents progression through G2/M and S cell cycle checkpoint for DNA repair before mitotic entry (Matheson et al., 2016a). Prior work has demonstrated that ectopic high-expression of WEE1 has been identified in several malignant tumors and associated with poor outcome, such as glioblastoma (Music et al., 2016), vulvar squamous cell carcinoma (Magnussen et al., 2013), ovarian carcinoma (Slipicevic et al., 2014), melanoma (Magnussen et al., 2012), and colorectal carcinoma (Egeland et al., 2016; Ge et al., 2017). On the contrary, in non-small cell lung cancer and colon cancer, patients with absence of WEE1 expression had poor prognosis (Yoshida, 2004; Cormanich et al., 2009). Until now, the expression and clinical significance of WEE1 in LSCC are still unknown. Here, we have investigated the expression and clinical significance of WEE1 in LSCC, and the anti-tumor effects and mechanisms of WEE1 inhibition against LSCC.

## Materials and Methods

### Patients and Specimens

A total 44 pairs of LSCC and corresponding adjacent normal tissues were obtained from patients who underwent partial or total laryngectomy without neoadjuvant radical or chemical therapy before and after surgery at the Department of Head and Neck, Cancer Center, Sun Yat-sen University (Guangzhou, China) from July 2008 to June 2015. The International Union against Cancer (UICC) 2002 norms for staging laryngeal carcinoma (clinical, endoscopic, and imaging) is strictly followed. Signed informed consents were obtained from all patients. The study was approved by the ethics committee of Sun Yat-sen University Cancer Center.

### Cell Culture and Reagents

Normal human bronchial epithelium cells HBEC, LSCC cells KB-3-1 and TU212 were cultured in Dulbecco’s modified Eagle’s medium (DMEM) with 10% fetal bovine serum (FBS), penicillin (100 U/ml), and streptomycin (100 ng/ml) at 37°C with 5% CO_2_ in a humidified incubator. MK-1775 was purchased from APExBIO (Shanghai). Methylthiazolyldiphenyl-tetrazolium bromide (MTT), propidium iodide (PI) and other chemicals were purchased from Sangon Biotech (Shanghai). *N*-acetyl-L-cysteine (NAC) and dihydroethidium (DHE) were purchased from Sigma-Aldrich. Anti-WEE1 (5285) and anti-pCDK T14/Y15 (28435) antibodies were from Santa Cruz Biotechnology. Anti-PARP (9542), anti-pHistone3 S10 (53348), and anti-C-Caspase3 (9964) were from Cell Signaling Technologies. Anti-γ-H2AX (AB55226) antibody was from Sangon Biotech. Anti-CDK1 (610037) antibody was from BD Biosciences. Anti-Ki-67 (2746-1) antibody was from Abcam. Anti-Vinculin (BM1611) antibody was from BOSTER Biological Technology.

### MTT Assay

Cells were seeded into a 96-well plate at a density of 0.5 × 10^4^ cells/well and treated with various concentrations of agents. After 3 days, MTT was added to each well at a final concentration of 0.5 mg/ml. After incubation for 4 h, the medium and MTT solution were removed from each well, and formazan crystals were dissolved in 100 μl of DMSO. Absorbance was measured at 570 nm by Multiscan Spectrum (Thermo Fisher). The data were analyzed by CompuSyn software with those results.

### Western Blot Assay

Cells were harvested and washed twice with cold PBS, then resuspended and lysed in RIPA buffer (1% NP-40, 0.5% sodium deoxycholate, 0.1% SDS, 10 ng/ml PMSF, 0.03% aprotinin, 1 μM sodium orthovanadate) at 4°C for 30 min. Lysates were centrifuged for 10 min at 14,000 × *g* and supernatants were stored at -80°C as whole cell extracts. Proteins were separated on 12% SDS-PAGE gels and transferred to polyvinylidene difluoride membranes. Membranes were blocked with 5% BSA and incubated with the indicated primary antibodies. Corresponding horseradish peroxidase-conjugated secondary antibodies were used against each primary antibody. Vinculin was used as a loading control. Signals were detected with the ChemiDoc XRS chemiluminescent gel imaging system (Analytik Jena).

### Cell Cycle Assay

Cells were harvested and washed twice with phosphate-buffered saline (PBS), then permeabilized with 70% cold ethanol for 2 h at 4°C. After washing twice in PBS, cells were resuspended with 0.5 ml PBS containing PI (50 μg/ml), 0.1% Triton X-100, 0.1% sodium citrate, and DNase-free RNase (100 μg/ml), and assessed by flow cytometry (FCM) (Beckman Coulter) after incubation at room temperature in the dark for 15 min. Fluorescence was measured at an excitation wavelength of 480 nm through a FL-2filter. Data were analyzed using ModFit LT 4.1 software.

### Cell Apoptosis Assay

Cells were harvested and washed twice with PBS, stained with Annexin V-FITC and PI in the binding buffer, and detected by FCM (Beckman Coulter) after 15 min incubation at room temperature in the dark. Fluorescence was measured at an excitation wave length of 480 nm through FL-1 (530 nm) and FL-2 filters (585 nm). The early apoptotic cells (Annexin V+/PI-) and late apoptotic cells (Annexin V+/PI+) were quantified.

### Reactive Oxygen Species Assay

Cells were incubated with 10 μM of DHE for 30 min at 37°C, and observed under fluorescence microscope (Olympus, Japan) immediately after washing twice with PBS. Five fields were taken randomly for each well. After photographed under florescent microscope, cells were rapidly digested, harvested and then washed twice with cold PBS, and detected by FCM (Beckman Coulter). The DHE Fluorescence intensity was measured and quantified at an excitation wave length of 518 nm through PE filters.

### Immunohistochemistry Assay

Formalin-fixed, paraffin embedded human LSCC tissues and KB-3-1 subcutaneous tumors in mice were stained with antibodies, respectively, using a microwave-enhanced avidin-biotin staining method. To quantify the protein expression, the following formula was used: immunohistochemical score = percentage of positive cells × intensity score. The intensity was scored as follows: 0, negative (no staining); 1, weak (light yellow); 2, moderate (yellow brown); and 3, intense (brown).

### Nude Mice Xenograft Assay

BALB/c nude mice were obtained from the Guangdong Medical Laboratory Animal Center and maintained with sterilized food and water. Five female nude mice with 5 weeks old and 16–18 g weight were used for each group. Every mouse was injected subcutaneously of the KB-3-1 cells (3 × 10^6^ in 100 μl of medium) under the right and left shoulders. When the subcutaneous tumors were approximately 0.3 cm × 0.3 cm (two perpendicular diameters) in size, the mice were randomized into two groups and taken orally with vehicle alone (0.5% methylcellulose) or MK-1775 (50 mg/kg) twice daily. The body weights of mice and the two perpendicular diameters (A and B) of tumors were recorded every day. The tumor volume (V) was calculated as:

$$\begin{array}{c} V=\pi /6{\left(1/2(A+B)\right)}^{3} \end{array}$$

The mice were anesthetized after experiment, and tumor tissue was excised from the mice and weighted. The rate of inhibition (IR) was calculated according to the formula:

$$\begin{array}{c} \mathrm{IR}=1-Mean tumor weight of experimental group/Mean tumor weight of control group\times 100\%. \end{array}$$

### Statistical Analysis

All statistical analyses were performed using the SPSS 20.0 statistical software package. Comparisons between two groups were performed using Student’s *t*-test or Mann–Whitney *U*-test, and comparisons among three groups were performed using one-way ANOVA or Kruskal–Wallis test. Fisher’s exact test or Pearson’s correlation were used to analyze the relationship between the expression levels of WEE1 protein. Kaplan–Meier method and the log-rank test were used to compare the survival of patients. ROC curve analyses were used to evaluate the prognostic ability. The difference in tumor volume between the two groups of mice was determined by repeated-measures analysis of variance. Data were presented as mean ± SD or median with the interquartile range. *P* < 0.05 was considered statistically significant.

## Results

### Up-Regulation of WEE1 Protein in LSCC Is Correlated With T Stages, Lymph Node Metastasis, Clinical Stages, and Poor Prognosis

**FIGURE 1:**
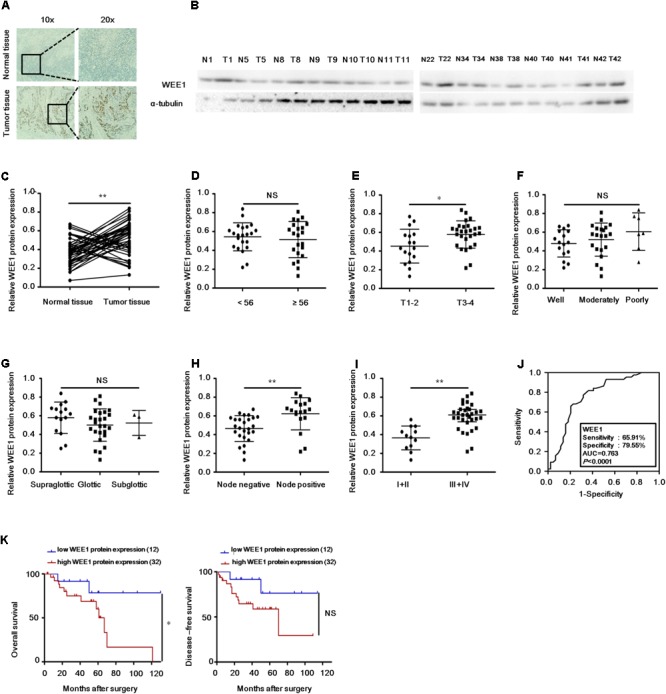
Up-regulation of WEE1 protein in LSCC is correlated with T stages, lymph node metastasis, clinical stages, and poor prognosis. **(A)** Immunohistochemistry analysis of WEE1 protein expression in the paired LSCC tissues and adjacent normal tissues. **(B)** Western blot analysis of WEE1 protein expression in the paired LSCC tissues and adjacent normal tissues. **(C)** Pearson’s correlation scatter plot of the expressions and WEE1 protein in human LSCC tissues. The relative WEE1 protein expression in LSCC tissues and adjacent normal tissues were analyzed with Student’s *t*-test. The relative WEE1 protein expression in two groups of LSCC tissues classified by age **(D)**, T stage **(E)**, lymph node metastasis **(H)**, and clinical stage **(I)** were analyzed with Student’s *t*-test. The relative WEE1 protein expression in three groups of LSCC tissues classified by differentiation **(F)** and primary location **(G)** were analyzed with one-way ANOVA. **(J)** ROC curve analysis of the discrimination between LSCC tissues and adjacent normal tissues by WEE1. **(K)** Kaplan–Meier analysis of overall survival and disease-free survival curves for LSCC patients with high or/and low expression of WEE1. Data are presented as mean ± SD or median with the interquartile range. ^∗^*P* < 0.05; ^∗∗^*P* < 0.01; NS, no statistical significance.

To investigate the expression and clinical significance of WEE1 in LSCC, the expression of WEE1 protein was detected in the total 44 pair LSCC and adjacent normal tissues. Immunohistochemical staining and Western blot results revealed that the expression of WEE1 protein was higher in LSCC tissues than adjacent normal tissues (**Figures 1A–C**). Furthermore, statistic analysis indicated that the expression of WEE1 protein was associated with T stage, lymph node metastasis and stage, but not with age, tumor grades and tumor primary locations (**Table 1** and **Figures 1D–G**). The expression of WEE1 protein in T1-2, negative lymph node metastasis and stage I+II groups were respectively lower than that in T3-4, positive lymph node metastasis and stage III+IV groups (**Figures 1E,H,I**). The levels of WEE1 protein could be a significant parameter to distinguish LSCC and adjacent non-tumorous tissues with an AUC of 0.763 (sensitivity = 65.91%, specificity = 79.55%; *P* < 0.0001) (**Figure 1J**). Moreover,

**Table 1:** Relationship between WEE1 protein expression level and clinicopathologic parameters.

Characteristics (*n*)	WEE1 protein level^a^	*p^b^*
**Age**		0.5880
<56 (22)	0.5436 ± 0.1482	
≥56 (22)	0.5155 ± 0.1915	
**T classification**		0.0103
T1-2 (17)	0.4529 ± 0.1807	
T3-4 (27)	0.5778 ± 0.1461	
**Differentiation**		0.5053
Well (16)	0.5479 ± 0.1970	
Moderately (21)	0.5082 ± 0.1179	
Poorly (7)	0.4728 ± 0.0858	
**Primary location**		0.3766
Supraglottis (15)	0.5793 ± 0.1680	
Glottis (26)	0.5015 ± 0.1734	
Subglottis (3)	0.5233 ± 0.1332	
**Lymph node metastasis**		0.0008
Negative (26)	0.4650 ± 0.1378	
Positive (18)	0.6228 ± 0.1716	
**Clinical stage**		<0.0001
I+II (12)	0.3650 ± 0.1267	
III+IV (32)	0.5913 ± 0.1411	

*^*a*^Scores determined by Western blot in mean ± SD. ^*b*^Student’s *t*-test (for two groups) or one-way ANOVA (for >2 groups).*

Kaplan–Meier analysis on the survival time of patients revealed that high WEE1 protein expression was correlated with poor overall survival, but not with disease-free survival (**Figure 1K**). Consequently, our results indicate that WEE1 may be a potential therapeutic target in LSCC.

### The WEE1 Inhibitor MK-1775 Suppressed the Growth of LSCC Cells *in vitro*

**FIGURE 2:**
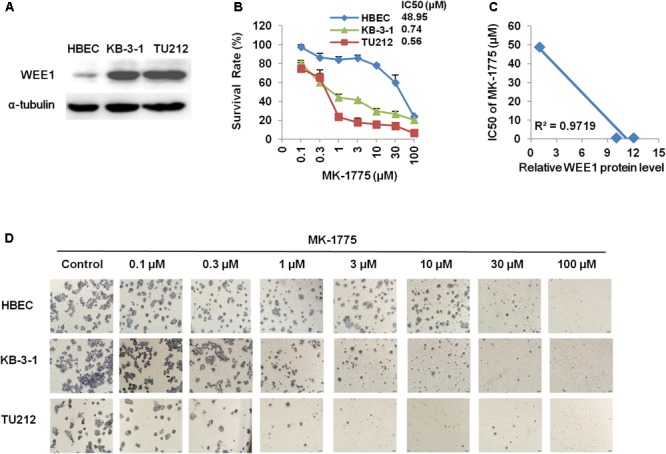
The WEE1 inhibitor MK-1775 suppressed the growth of LSCC cells *in vitro*. **(A)** Western blot analysis of WEE1 protein expression in KB-3-1, TU212, and HBEC cells. Cells were treated with the indicated concentration of MK-1775 for 72 h. Cell survival was measured by MTT assay. The representative growth curves of KB-3-1, TU212, and HBEC cells treated with MK-1775 **(B)**, correlation analysis of MK-1775 IC_50_ values and relative WEE1 protein levels **(C)**, and images of cells stained with MTT for 4-h **(D)** are shown.

To further explore whether WEE1 is a therapeutic target in LSCC, we examined the protein expression of WEE1 and cytotoxicity of a WEE1 inhibitor MK-1775 in LSCC cells. Two human LSCC cell lines KB-3-1 and TU212 expressed higher level of WEE1 protein than one normal bronchial epithelium cell line HBEC (**Figure 2A**). Then these cells were treated with the increasing concentrations of MK-1775 for 72 h. As shown in **Figures 2B,D**, MK-1775 inhibited the growth of LSCC cells in a dose-dependent manner with the IC_50_ values of 0.74 μM and 0.56 μM in KB-3-1 and TU212 cells respectively. However, the IC_50_ value of MK-1775 in HBEC cell is 48.95 μM, suggesting MK-1775 is significantly more cytotoxic to LSCC cells than normal cells. Moreover, the IC_50_ values of MK-1775 in these three cells were negatively correlated with their WEE1 protein levels (**Figure 2C**).

### MK-1775 Induced Cell Cycle Arrest and Apoptosis in LSCC Cells

**FIGURE 3:**
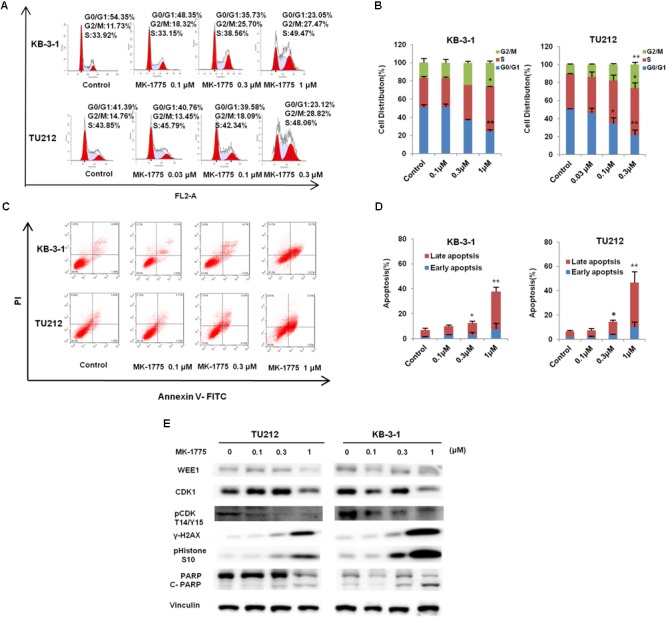
MK-1775 induced cell cycle arrest and apoptosis in LSCC cells. KB-3-1 and TU212 cells were treated with the indicated concentrations of MK-1775 for 48 h, then cell cycle and apoptosis were detected by FCM. The protein expression was examined by Western blot after lysing cells. The representative charts **(A,C)**, quantified data **(B,D)** and Western blot results **(E)** of three independent experiments are shown. ^∗^*P* < 0.05 and ^∗∗^*P* < 0.01 vs. corresponding control.

To determine whether the growth inhibition was due to cell cycle arrest, cell cycle distribution was examined after MK-1775 treatment. KB-3-1 and TU212 cells were treated with the incremental concentrations of MK-1775 for 48 h, stained with PI and examined by FCM. As shown in **Figures 3A,B**, MK-1775 induced the accumulation in S and G2/M phase and reduction in G0/G1 phase in these two cell lines. Next, the related proteins of cell cycle were detected by Western blot. Treatment with MK-1775 at 1 μM for 48 h downregulated the protein expressions of WEE1, CDK1, and pCDK T14/Y15, but upregulated the protein expression of γ-H2AX and pHistone3 S10 (**Figure 3E**).

To further determine whether MK-1775 could induce cell apoptosis in LSCC cells, TU212 and KB-3-1 cells were treated with 0.1, 0.3, 1 μM of MK-1775 for 48 h, stained with Annexin V/PI and examined by FCM. As shown in **Figures 3C,D**, MK-1775 dose-dependently induced early apoptosis (Annexin V+/PI-) and late apoptosis (Annexin V+/PI+) in both cells. Furthermore, the protein levels of apoptosis marker cleaved-PARP was increased in a dose-depend manner after MK-1775 treatment in both cells (**Figure 3E**).

### MK-1775 Enhanced the Intracellular ROS Levels in LSCC Cells

**FIGURE 4:**
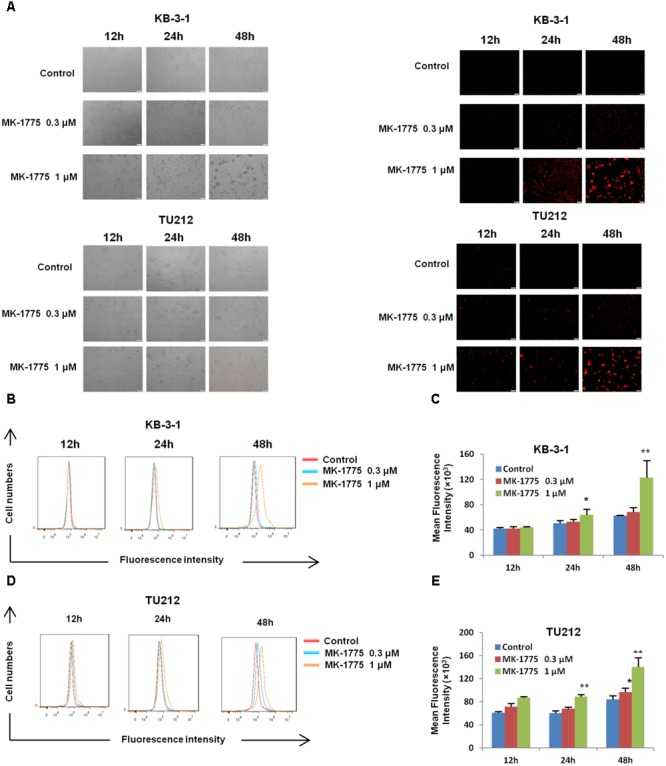
MK-1775 enhanced the intracellular ROS levels in LSCC cells. KB-3-1 and TU212 cells were treated with MK-1775 at the indicated times and concentrations then stained with DHE, photographed and quantified respectively under florescent microscope and FCM. The representative micrographs **(A)**, red fluorescent intensity charts by FCM **(B,D)** and quantified results of red fluorescence **(C,E)** of KB-3-1 and TU212 cells in three independent experiments were shown. ^∗^*P* < 0.05 and ^∗∗^*P* < 0.01 vs. corresponding control.

There was increasing evidence that extravagant production of ROS was relevant to carcinogenesis, malignant behavior, and mitochondria-mediated apoptosis (Chung, 2016; Miyata et al., 2017; Prasad et al., 2017), and cancer cells generally have higher ROS levels than their normal counterparts (Chung, 2016; De Sa Junior et al., 2017). Thus, we speculated that MK-1775 caused apoptosis in LSCC cells in relation to excessive ROS generation. Firstly, the cellular ROS was tagged by DHE fluorescence staining in MK-1775-treated cells. As illustrated in **Figures 4A–E**, MK-1775 enhanced the detectable red fluorescent signals of DHE in both KB-3-1 and TU212 cells in the concentration-and time-dependent manners, indicating the intracellular ROS levels were increased after MK-1775 treatment.

### NAC Impeded MK-1775-Induced ROS Production and Cell Apoptosis

**FIGURE 5:**
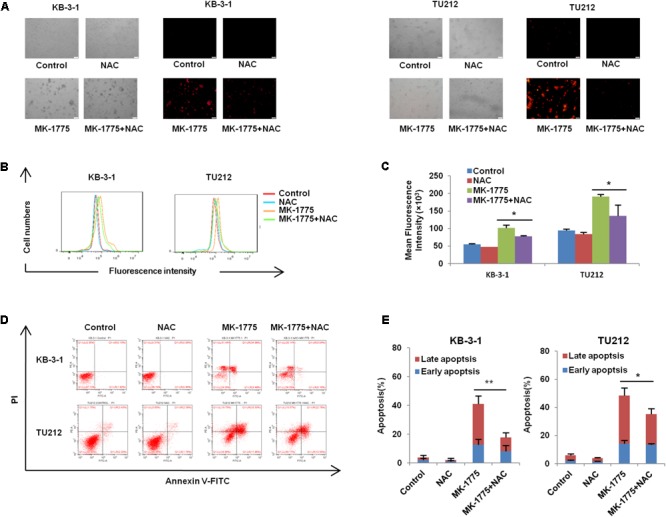
NAC impeded MK-1775-induced ROS production and cell apoptosis. KB-3-1 and TU212 cells were treated with 1 μM MK-1775 for 48 h in the presence or absence of 5 mM NAC pretreatment for 1 h, then stained with DHE, photographed and quantified respectively under fluorescent microscope and FCM. The apoptosis was detected by FCM with Annexin V/PI staining. The representative micrographs **(A)**, red fluorescent intensity charts by FCM **(B)** and quantified results of red fluorescence **(C)**, the apoptosis charts **(D)** and quantified results **(E)** of three independent experiments were shown. ^∗^*P* < 0.05 and ^∗∗^*P* < 0.01 vs. corresponding control.

To investigate whether ROS generation was involved in the MK-1775-induced cell apoptosis, a specific ROS scavenger (NAC) was co-treated with MK-1775 in both KB-3-1 and TU212 cells. MK-1775-induced DHE fluorescent signals were attenuated by NAC in both cells (**Figures 5A–C**). Moreover, a reduction of cell apoptosis after co-treatment with MK-1775 and NAC was observed compared with MK-1775 treatment alone in both cells (**Figures 5D,E**). Collectively, these results suggest that NAC impedes MK-1775-induced ROS production and cell apoptosis.

### MK-1775 Inhibited the Growth of KB-3-1 Xenografts in Nude Mice

**FIGURE 6:**
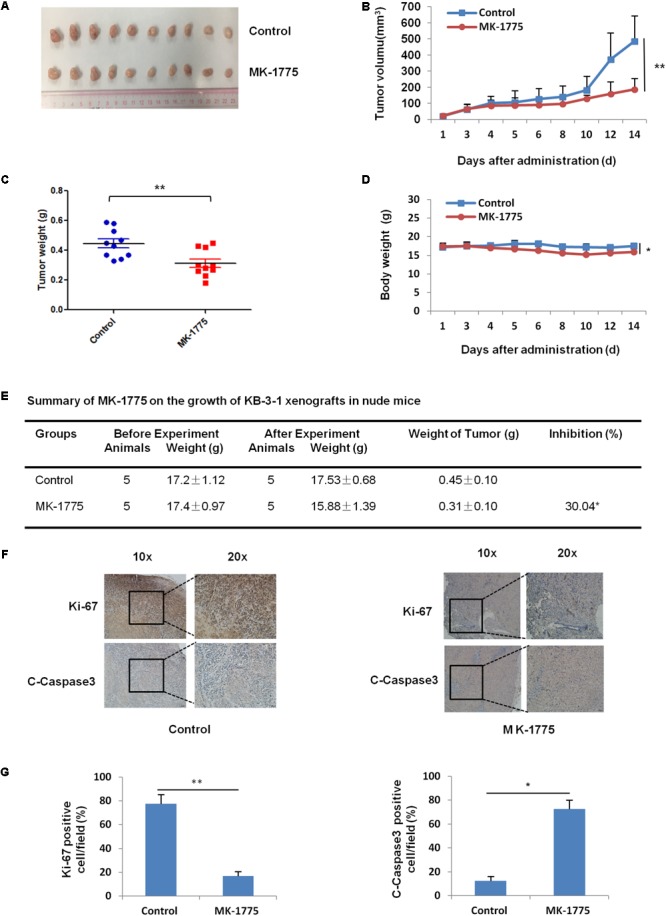
MK-1775 inhibited the growth of KB-3-1 xenografts in nude mice. Each mouse was injected subcutaneously with KB-3-1 cells (3 × 10^6^ in 100 μl of medium) under the right and left shoulders. When the subcutaneous tumors were approximately 0.3 cm × 0.3 cm^2^ (two perpendicular diameters) in size, mice were randomized into two groups, and were taken orally with vehicle alone (0.5% methylcellulose) or MK-1775 (50 mg/kg) twice daily. The body weights of mice and tumor volume were recorded. The mice were anesthetized after experiment, and tumor tissue was excised from the mice and weighted. The original tumors **(A)**, tumor volume **(B)**, tumor weight **(C)**, body weight **(D)**, summary data **(E)**, immunohistochemical staining of Ki-67 and C-caspase3 in subcutaneous tumors **(F)** and quantified of Ki-67 and C-caspase3 positive cells **(G)** were also shown. The values presented are the means ± SD for each group. ^∗^*P* < 0.05 and ^∗∗^*P* < 0.01 vs. corresponding control.

To confirm the antitumor effects of MK-1775 *in vivo*, KB-3-1 subcutaneous xenografts were generated in the nude mice. As shown in **Figures 6A–E**, treatment with MK-1775 inhibited the growth of KB-3-1 xenografts with the inhibition ratio of 30.04% by reducing the tumor volumes and weights. Furthermore, mice body weight in MK-1775 group was lower than that of control group, suggesting that MK-1775 at the indicated dose did cause toxicity in mice (**Figure 6D**). Additionally, the results of immunohistochemical staining showed that the percentage of proliferation marker Ki-67 positive cells was decreased, while apoptosis marker cleaved-caspase 3 positive cells was increased in KB-3-1 xenografts after MK-1775 treatment (**Figures 6F,G**).

## Discussion

In this study, we reported that WEE1 was significantly expressed in LSCC tissues than adjacent normal tissues, and the expression level of WEE1 was associated with T stage, lymph node metastasis and poor survival of patients with LSCC. These data are similar with previous studies which have shown that WEE1 has served a crucial role in tumorigenesis and related with poor prognosis in several cancers that harbor WEE1 amplifications (Magnussen et al., 2012, 2013; Slipicevic et al., 2014; Music et al., 2016). Therefore, targeting WEE1 has emerged as a promising therapy for human cancers (Matheson et al., 2016a; Geenen and Schellens, 2017). MK-1775, a first-in-class small-molecule inhibitor of WEE1 with undergoing clinical evaluation, abated phosphorylation of CDK1 at Tyr15 and caused mitotic catastrophe in cancer cells (Kreahling et al., 2013). Early preclinical studies suggested MK-1775 was capable of abrogating the G2/M checkpoint allowing for premature entry into mitosis to exert a toxic effect specifically in p53 deficient tumor cells (Guertin et al., 2013; Hirai et al., 2014). Our results showed that inhibition of WEE1 with MK-1775 caused deceased viability, cell cycle arrest, and cell apoptosis, suggesting WEE1 was essential for cell proliferation and tumorigenicity in LSCC cells. MK-1775 has been reported that sensitizes cancers of head and neck (Osman et al., 2015), brain (Matheson et al., 2016b), and non-small cell lung cancer (Richer et al., 2017) to the DNA-damage drug cisplatin as well as pancreatic cancer cells (Kausar et al., 2015) to gemcitabine. Additionally, MK-1775 has promising synergistic antitumor effect when combined with CHK1 inhibitors LY2603618 and Sirt1 inhibitor Ex527 in various malignancies (Chen et al., 2017; Hauge et al., 2017), suggesting a novel strategy for MK-1775-mediated cancer treatment.

Biological roles of ROS were intricate and contradictory in cancers (Halliwell, 2013). Under a low or moderate increase, ROS is vital for regulating various cell physiological processes and maintaining cellular homeostasis, which functions as signaling molecules favoring tumorigenesis (Miyata et al., 2017). On the contrary, exorbitant production of ROS acts to cause DNA damage and oxidative stress to engender genotoxic or even proapoptotic and autophagic effect on cancer cells (Trachootham et al., 2009; Schieber and Chandel, 2014; Kruk and Aboul-Enein, 2017). Accordingly, this oxidative stress causing the cumulative effects may induce cancer cells susceptible to chemotherapeutic agents treatment that perform by amplifying ROS generation (Schumacker, 2006). ROS goes up when cells prematurely enter mitosis, and that the increased ROS drives cell death (Marchetti et al., 2006). To confirm that this was occurring we detected the intracellular level of ROS in MK-1775-treated LSCC cells, we found ROS significantly increased following Wee1 inhibition, and we could limit death by reducing ROS with NAC.

## Conclusion

Our findings suggest that WEE1 is a potential therapeutic target in LSCC, and inhibition of WEE1 is the prospective strategy for LSCC therapy.

## Author Contributions

M-LY, PL, JY and ZS designed the experiments, performed the experiments, analyzed the data, and wrote the paper. Z-HX, J-MD, HL, A-KY, X-JL, Q-WJ, YY, J-RH, KW, M-NW, and YL performed the experiments. All authors read and approved the final manuscript.

## Conflict of Interest Statement

The authors declare that the research was conducted in the absence of any commercial or financial relationships that could be construed as a potential conflict of interest.
